# Foods Department of the Local Government Board

**Published:** 1913-09-20

**Authors:** 


### The Hospital

The Workers' Newspaper of Administrative Medicine and Institutional Life, Administration, National Insurance and Health.

No. 1421 Vol. LIV. SATURDAY, SEPTEMBER 20, 1913. PRICE ONE PENNY

### FOODS DEPARTMENT OF THE LOCAL GOVERNMENT BOARD.

At every stage of human development the question of food and its preparation has been one of paramount importance, and has formed a subject of never-failing interest; in each decade there are changes, even fashions, in food. As we may imagine our cave-dwelling ancestors to have discussed the succulence of the varieties of marrow and the digestibility of fermented nuts, so to-day we constantly hear views (probably often little more illuminating or conclusive) concerning vegetarianism, the purin-free dietary, and the value of alcohol. In this restless and crowded age, harassed as we are by dietetic opinions of doubtful wisdom and dietetic advertisements of more than doubtful veracity, alimentary perfection is sought in vain. The subject of dietary is still a complex, difficult, and, to many, a fascinating one. The alteration in the conditions—some would say the decay—of home life; the scarcity of capable cooks; the engrossing requirements of work and, even more often, of pleasure; the increased cost of living; these and other factors have a bearing upon the matter. Upon many points we seek enlightenment: we want to know, for instance, whether "English" meat has often any claim to that title beyond that of having been killed at Deptford or Liverpool; whether the dairy-chemist exists for the benefit of the public; whether naturally ripened cheese is obtainable; whether jam consists of fruit and sugar; and whether "sweet" oil has any relationship to olives. With much justification it may be feared that we are tending to abandon simple fresh animal and vegetable foods and are becoming increasingly dependent upon preserved, artificially prepared, or chemically dressed foods and upon food substitutes and food adjuvants.

With much interest and some curiosity, therefore, we turn to the Report of the Government Chemist upon the work of the Government Laboratory for the year ended March 31 last. Far reaching as is the range of his activities we shall find information in the Report upon few such points as we have mentioned. It reflects in but a limited degree the unobtrusive work of the Foods Department of the Local Government Board and of the various health authorities, inspectors, and analysts. Though rarely illuminated in the public press, their work is very real, increasing, and important. In the safeguarding of the nation's food supply much has now to be done which does not come within the scope of the chemical laboratory.

The Government Chemist carries out examinations for some eighteen Departments and public bodies, including the Board of Customs and Excise, the Local Government Board, the Post Office, the War Office, and the Agricultural Departments. In the cold light of statistics his Report concerns, including those examined in provincial stations, 354,759 samples; the number being considerably in excess of that dealt with in the previous year. The character of these samples is so diverse as to include, amongst other things, methylated spirits, water, tallow, paints, cycle tyres and tubes, pharmaceutical preparations, sheep-dips, fertilisers, matches, and tobacco. The laboratory is even called upon to furnish an opinion as to the probable date at which were made entries produced as evidence of the age of applicants for old-age pensions. In six documents examined during the year the writing (submitted as proof on the strength of antiquity) was found to be of recent date or to have been tampered with. Adhesive substances used in the illicit removal of letters from postal boxes were examined, as were obliterating fluids used by the postal authorities; there is, however, no record of the composition of "obliterating" or other fluids placed in letter-boxes by suffrage-seeking enthusiasts.

The food-stuffs dealt with consist largely of imported and dutiable articles, those containing alcohol assuming a considerable importance. Within this class fall, somewhat strangely, beer-substitutes and other "temperance" beverages; in these the legal limit of 2 per cent. of proof spirit is not infrequently exceeded, ten samples of ginger-beer or herb-beer being found to contain percentages varying from 5.7 to 11.5 and constituting decidedly stimulating forms of refreshment. Some non-alcoholic ciders are real ciders diluted by the addition of sugar solutions, but many are entirely free from fermented apple-juice and are simply solutions of sugar which have been aerated, flavoured, and coloured. Imported liqueur chocolates, of which 269 samples were examined, contain an average percentage of 5.4 of proof spirit, the highest amount being 21.9.

It is interesting to note that 224 samples of tobacco, amongst those tested for moisture-content, were of home-grown leaf; and that, after a lapse of nearly forty years, the manufacture of sugar from beetroot was recommenced in this country last autumn at North Cantley, in Norfolk. All consignments of tea are inspected at the port of entry, and samples are more fully examined if it be thought necessary. A few were condemned as containing sand or other foreign matter, or as being unfit for human consumption; no evidence of intentional adulteration was found. The rejected tea is denatured, and from it theine, for use as a drug, is extracted. The coffee substitutes found were of the usual character, consisting, apart from chicory, of caramel, roasted cereals and figs; some chicory is grown in this country. Fresh milk, described as "pasteurised," was imported in churns between November and February. Much "condensed" milk, from which water has been removed by evaporation and to some of which sugar has also been added, is examined. "Of late years condensed milk has been prepared in which the removal of the water has been carried much further, leaving the milk-solids in a nearly dry condition. This product, described as dried milk or milk-powder, is prepared both from whole and from skimmed milk and contains from 2 to 8 per cent. of water; an increasing use is being made of it. The requirement, under the provisions of the Food and Drugs Act, that packages of this preparation made from separated or skimmed milk shall be labelled accordingly is in numerous instances not yet complied with. Many of the examinations of dried milk were made in connection with an inquiry instituted by the Local Government Board. The results of the regulations recently issued by that Board as to the use of preservatives in milk, cream, etc., are highly satisfactory. The information as to the country of origin of milk and milk-products is incomplete; Swiss milks are imported from France and Swedish milks from Denmark; butter from Russia and Siberia is imported through Denmark. The importation of butter from Canada and the United States is diminishing, whilst from Australia and New Zealand it appears to be increasing.

Many tests were in connection with industrial diseases and matters affecting the health of factory workers. We find mention of pottery-glazes, "file-cutters' beds" (in which the permissible quantity of lead is 5 per cent.), and the dust produced by electric arc-lamps. This dust is said to contain carbon, silica, and sulphates of sodium and potassium. The Report impresses us as a simple record of extraordinarily varied and valuable work, which is largely unappreciated by the institutional world that benefits indirectly so much from it.

